# Understanding teachers’ technostress creators due to IT use at work: a mixed-methods study

**DOI:** 10.3389/fpsyg.2026.1887403

**Published:** 2026-10-02

**Authors:** Ni Yao, Qiong Wang

**Affiliations:** 1 School of Education Science, Nanjing Normal University of Special Education, Nanjing, China; 2 College of Science, Shaoyang University, Tangdukou, China

**Keywords:** IT use behaviors, mixed-method design, teachers, technostress appraisal, technostress creators

## Abstract

With the digitization of education, the use of information technology (IT) at work has become a requirement for teachers, rather than an option. However, IT is a double-edged sword that brings teachers great benefits and additional challenges (e.g., technostress). The present study employed a mixed-methods design to understand context-specific technostress creators and their associations with cognitive appraisal outcomes and IT use behaviors among teachers. Phase 1 identified six technostress creators—unreliability (UNR), role conflict, teaching interruption (TI), work overload (WO), information overload (IO), and technology integration (TIG)—through semi-structured interviews with 20 teachers. Phase 2 was conducted to empirically examine how technostress creators influence teachers’ IT use behaviors (i.e., innovative use [IU] and avoidant use) through cognitive appraisal outcomes. The results from structural equation modeling involving 265 Chinese teachers demonstrated that teachers tend to appraise unreliability, role conflict, and teaching interruption as threats, but appraise work overload and technology integration as challenges. Moreover, challenge appraisal is likely to stimulate innovative IT use and decrease avoidant IT use, while threat appraisal (TA) increases avoidant IT use. The findings extended the literature related to IT research by identifying context-specific technostress creators and linking them to IT use among teachers.

## 1 Introduction

With the increasing digitization of education worldwide, the use of information technology (IT) has become a routine part of teachers’ work (Nascimento et al., 2024). IT offers numerous benefits to teachers, including increased flexibility and mobility, enhanced productivity, detailed feedback, and improved interactions and assessments (Liu et al., 2024). However, like a coin with two sides, IT also has positive and negative aspects. K-12 teachers, in particular, encounter additional challenges, such as technostress, which refers to the stress resulting from using IT at work (Tarafdar et al., 2007). Research has consistently revealed that technostress represents the negative aspects of IT, resulting in various undesirable consequences (Chou and Chou, 2021; Jena, 2015). Since individuals overexposed to IT are at high risk of experiencing technostress, it is important to understand the technostress experienced by teachers, especially as education increasingly incorporates diverse emerging technologies (Lee et al., 2024).

Technostress creators, or techno-stressors, refer to events or stimuli that trigger technostress (Maier et al., 2022). Initially, Tarafdar et al. (2007) identified five typical technostress creators, including techno-overload, techno-complexity, techno-invasion, techno-insecurity, and techno-uncertainty, based on their research involving IT employees. Since then, numerous scholars from a wide range of professional disciplines have explored individuals’ technostress from the perspective of these five technostress creators (Al-Ansari and Alshare, 2019; Özgür, 2020; Upadhyaya and Vrinda, 2021; Zhao et al., 2020). However, recent research has shown that technostress is context-specific, and technostress creators differ across disciplines (Shi et al., 2024). Understanding context-specific technostress creators in a given IT use situation is crucial for researchers and practitioners to effectively address technostress (Nastjuk et al., 2023).

In the educational field, studies on teachers’ technostress have been limited to evaluating the five typical technostress creators initially identified in the IT industry within European nations, often using quantitative methods (e.g., Dong et al., 2020). However this approach overlooks the variation in technostress creators that may arise from espoused cultural values and the specific context of IT use (Krishnan, 2017). Such a narrow focus could impede our understanding of the underlying technostress creators in educational contexts. Since some studies (e.g., Zhao et al., 2020) have reported that individuals’ appraisal processes have a great impact on the consequences of technostress creators, more attention should be paid to teachers’ cognitive assessments. Furthermore, while existing studies have identified and examined several consequences of technostress creators, there has been little research on how these stressors impact teachers’ use of IT, especially in terms of innovative use [IU] and avoidant use, through teachers’ appraisal processes.

To bridge these gaps, this study aimed to gain an in-depth understanding of teachers’ technostress using a mixed-methods design. In Phase 1, a qualitative method was used to identify the technostress creators associated with IT use among teachers. In Phase 2, a quantitative method was employed to investigate how technostress creators influence behavioral intentions for IT use. We formulated a conceptual research model based on the transactional theory of stress (Lazarus and Folkman, 1984) and tested it with empirical data. The details of the research design are shown in Figure 1.

**Figure 1:**
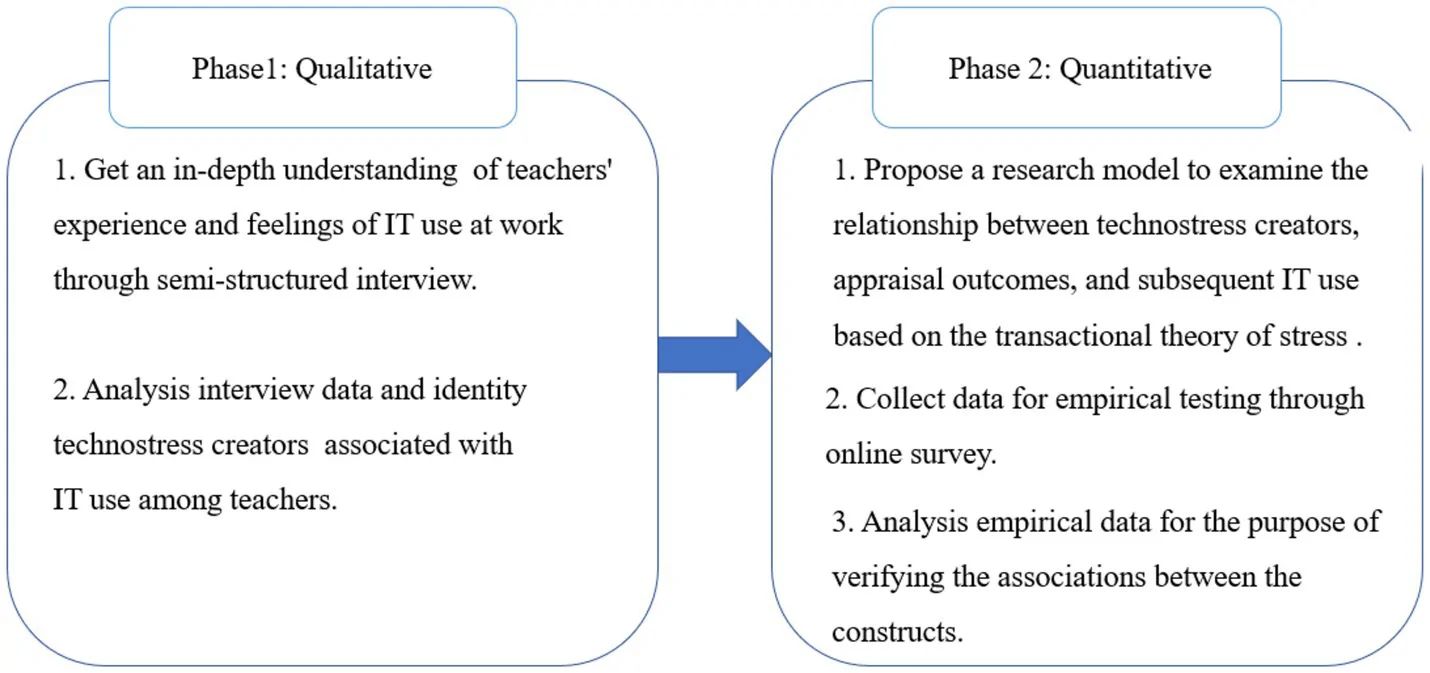
Design of the research.

## 2 Theoretical basis

### 2.1 Technostress and technostress creators

Technostress describes the stress resulting from technological demands at work (Ayyagari et al., 2011; Tarafdar et al., 2019). In the late 20th century, some scholars became concerned and attempted to conceptualize technostress (e.g., Brod, 1984). Subsequently, scholars empirically and conceptually explored technostress associated with employees who have to use IT intensively in their work and published their studies in top-tier information systems (IS) journals (e.g., Ayyagari et al., 2011; Califf et al., 2020; Maier et al., 2022; Tarafdar et al., 2007). The majority of scholars agree that technostress is an undesirable side of IT use, leading to negative psychological and behavioral consequences such as anxiety, burnout, decreased productivity and well-being, and increased turnover intention and discontinuous IT use (Chou and Chou, 2021; de Oliveira Malaquias and de Souza Júnior, 2023; Jena, 2015; Khedhaouria and Cucchi, 2019; Nastjuk et al., 2023; Suh and Lee, 2017). Technostress creators, also termed techno-stressors, were initially introduced by Tarafdar et al. (2007) and refer to the stimuli or causes (e.g., characteristics of IT and events associated with IT) that have the potential to create technostress (Ayyagari et al., 2011; Nastjuk et al., 2023). As IT use has become an indispensable part of work, users inevitably encounter technostress creators (Tarafdar et al., 2019). Tarafdar et al. (2007) introduced five technostress creators: techno-complexity, techno-overload, techno-invasion, techno-insecurity, and techno-uncertainty. In particular, techno-complexity refers to individuals having to spend extra time and energy on improving their technology literacy; techno-overload refers to individuals having to work for longer hours to adapt to the requirements of new technologies; techno-invasion refers to individuals’ personal lives being invaded by work due to the continuous connectivity of IT; techno-insecurity refers to individuals’ anxiety about the risk of being replaced by emerging technologies or people who are proficient in technologies; and techno-uncertainty refers to individuals’ perception of uncertainty resulting from the frequent updating of technology. Although some other studies identified additional technostress creators such as technological workplace surveillance (Dragano and Lunau, 2020) and privacy issues (Ayyagari et al., 2011), these five typical technostress creators were the most widely recognized and adopted by IS scholars (Dahabiyeh et al., 2022).

However, some scholars argued that technostress creators may also vary because the work methods and commonly used IT tools differ among industries (Maier et al., 2022; Nastjuk et al., 2023). For instance, Shi et al. (2024) established five technostress creators (i.e., persistence, role conflict, work connectivity, visibility, and emotional interruption) through qualitative analysis of hospitality employees’ use of enterprise social media. Maier et al. (2022) summarized eight technostress creators in the context of trial-period IT use from three aspects. We summarized and compared the typical studies related to technostress creators and clarified their limitations for teachers (Appendix A). Thus, there is a need to explore the underlying technostress creators from the perspectives of specific disciplines.

### 2.2 Teachers’ technostress and creators

The ongoing digitalization of education has led to the emergence of technostress among teachers (Dong et al., 2020; Estrada-Muñoz et al., 2020). Al-Fudail and Mellar (2008) initially investigated the nature of teachers’ technostress in technology-rich classrooms. Then, a growing number of scholars have conducted studies on the technostress of teachers at different educational levels, with the antecedents and consequences of technostress being the most frequently studied topics. The literature suggests that teachers’ (a) demographic characteristics such as age, gender, and IT use experience (Marchiori et al., 2019), (b) personal resources such as Technological Pedagogical Content Knowledge (TPACK) (Özgür, 2020), IT self-efficacy (Dong et al., 2020), and (c) environmental factors such as school support (Joo et al., 2016) are closely linked to their technostress.

Several scholars have studied the five typical technostress creators (i.e., techno-complexity, techno-overload, techno-invasion, techno-insecurity, and techno-uncertainty) among teachers. As shown in Table 1, these studies can be categorized into two types: some studies considered technostress creators as sub-dimensions of technostress that affect teachers at an aggregate level (Jena, 2015; Özgür, 2020), resulting in negative outcomes; others treated technostress creators individually and reported that the impacts of technostress vary (de Oliveira Malaquias and de Souza Júnior, 2023; Li and Wang, 2021). For instance, Dahabiyeh et al. (2022) reported that, for teachers’ productivity, techno-invasion impacts negatively, while techno-insecurity and techno-uncertainty impact positively; techno-overload and techno-complexity are insignificant. Furthermore, findings about the impact of a given technostress creator remain inconsistent. Taking techno-overload, for example, it was expected to exert a negative impact on work performance based on the literature (e.g., Tarafdar et al., 2007), but some researchers (e.g., Li and Wang, 2021) found that it significantly and positively predicts teachers’ performance, while others found its impact insignificant (e.g., Dahabiyeh et al., 2022). The varying effects of technostress creators illustrate that technostress has a double-edged nature, whereas the inconsistent findings call for further study to gain a deeper understanding of teachers’ technostress.

**Table 1:** Typical studies associated with teachers’ technostress creators.

Author	Method	Sample	Technostress creators	Consequences
Estrada-Muñoz et al. (2022)	Questionnaire	567 kindergarten directors	Considered aggregately	Skepticism, fatigue, anxiety, inefficacy
Jena (2015)	Questionnaire	216 academicians	Considered aggregately	Commitment, job satisfaction, negative affectivity, technology-enabled performance
Özgür (2020)	Questionnaire	349 in-service high school teachers	Considered aggregately	–
Li and Wang (2021)	Questionnaire	312 university teachers	Considered individually	Work performance.
Mingnon and Kyu Yon (2020)	Questionnaire	164 teachers	Considered individually	Job satisfaction, teacher efficacy
Wang and Zhao (2023)	Questionnaire	289 K-12 teachers	Considered individually	Attitudes toward information and communication technology (ICT), ICT adoption intentions
de Oliveira Malaquias and de Souza Júnior (2023)	Questionnaire	372 teachers	Considered individually	Burnout, job satisfaction
Califf and Brooks (2020)	Questionnaire	416K-12 teachers	Considered individually	Burnout, turnover intention
Yang and Lee (2021)	Questionnaire	210 childhood teachers	Considered individually	TPACK
Dahabiyeh et al. (2022)	Questionnaire	217 university teachers	Considered individually	Online teaching exhaustion, teaching staff productivity

Technostress creators include techno-complexity, techno-overload, techno-invasion, techno-insecurity, and techno-uncertainty.

## 3 Phase 1: Identify technostress creators of teachers’ IT use

### 3.1 Sample and procedure

To obtain an in-depth understanding of teachers’ experiences and perceptions about IT use at work, we performed semi-structured interviews with 20 teachers from southern China. The sample comprised 13 (65%) females and 7 (35%) males, whereas 1 (5%) was from kindergarten, 5 (25%) were from primary schools, 6 (30%) were from middle schools, and 8 (40%) were from universities. The average age was 34 (SD = 8.76), with an average of 10 years of teaching experience (SD = 8.81). For all these interviewees, using IT tools is a regular part of work. Before the interview, we informed every participant of our research purpose and obtained their written consent. The sample was recruited through maximum-variation purposive sampling, covering teachers across different educational stages, ages, and genders. Although this non-probabilistic sampling strategy does not support statistical representativeness for the broader teacher population, the diverse sample enabled us to capture the heterogeneous experiences of workplace IT-use-related technostress among teachers. The demographic distribution of the interviewees is shown in Appendix B.

In the interview process, the researchers conducted in-depth interviews with teachers around the pre-designed interview outline (Appendix C), encouraging teachers to provide detailed descriptions of their feelings about IT use. Additionally, flexible guidance and questioning were used to explore the core issue (i.e., what characteristics of IT or events associated with IT do you think contribute to such stress?) to elicit possible unexpected ideas/opinions. The interviews lasted 25 and 40 min and were recorded and transcribed for the subsequent qualitative analysis.

### 3.2 Qualitative analysis and results

The interview data were analyzed following the coding guidelines with reference to Corbin and Strauss (2014) to identify the technostress creators associated with teachers’ IT use. In the qualitative analysis process, two researchers independently coded all interview transcripts. Discrepant codes were discussed iteratively until consensus was reached. However, respondent validation could not be performed because the interview participants could no longer be contacted after data collection. First, we performed open coding to conceptualize the raw data. Then, we categorized 15 subcategories by combining similar codes identified in the open coding stage. The aggregation from 15 subcategories to higher-order conceptual units followed three decision rules: (a) Semantic similarity rule: subcategories with highly overlapping core meaning and descriptive content were candidates for merging; (b) Theoretical-grounding rule: the merged categories needed to align with existing technostress literature while still preserving unique contextual features observed among teachers; (c) Distinctiveness rule: Each resulting higher-order conceptual unit must be conceptually distinguishable from the others.

Finally, we identified six conceptual units: unreliability (UNR), role conflict, teaching interruption (TI), information overload (IO), work overload (WO), and technology integration (TIG). The results of the interview data analysis are shown in Table 2.

**Table 2:** Coding results of the interview data.

Category	Subcategory	Examples of open coding results
Unreliability	System failures	Sometimes the teaching equipment cannot be started for unknown reasons.
		Before peer observation, I always worry about whether the IT tools can run smoothly.
	System breakdowns	Sometimes the teaching system halts unexpectedly.
		Encountering system errors in class made me feel embarrassed.
	Slow system response	The teaching system responds slowly when too many programs are running simultaneously.
		The online teaching platforms may not function properly during peak hours.
Role conflict	Multiple work demands	With IT tools, I can handle multiple tasks at the same time.
		I often respond to students’ questions online while doing housework at home.
		I usually need to design teaching plans and develop corresponding teaching support materials.
	Capability-exceeding job demands	It is not easy for me to develop high-quality PowerPoint slides for my teaching.
		It’s difficult to fulfil the IT-related requirements at work.
		It’s frustrating when I have to cope with technological problems.
Teaching interruption	IT-caused teaching interruptions	Sometimes my instructional presentations fail due to incompatibility, and I have to change the teaching plan temporarily.
		When the duration limit is reached, the ongoing live teaching will be suspended.
	IT-related teaching distractions	I cannot focus on teaching activities when I have to operate unfamiliar teaching systems or devices.
	Unexpected information intrusions	My thoughts during teaching are easily interrupted and disturbed by calls and messages.
		The continuous connectivity of smartphones enables teaching interruption at any time.
		Sometimes teaching activities are disrupted by unexpected information popping up on the screen.
Information overload	Massive information inflows	I joined many work-related social network groups.
		There is often a lot of unread work-related information on smartphones.
		By checking the enormous amount of information in the work-related social network groups, I can keep up with work dynamics.
		Almost all work notifications and requirements will be posted in the work-related social network groups.
		Sometimes, the amount of information on smartphones exceeds my processing capabilities.
	Irrelevant information inputs	Of the enormous amount of information, much of it is irrelevant to my work.
		I mark or save important information promptly in order to avoid having it buried by other information.
Work overload	Additional work burdens	When new technologies are introduced into work, I have to spend time familiarizing myself with them.
		I am a bit resistant to the new platforms introduced because I need to spend time adapting to them.
	Repetitive work tasks	I often need to repost the homework assigned in class groups to ensure that parents are aware of it.
		I have to frequently use IT tools to complete a lot of administrative tasks such as grading and attendance.
		Sometimes, I have to work on different applications or platforms to fulfil a task, resulting in repetitive work.
	Time-consuming work tasks	It is time-consuming to prepare digital teaching materials.
		The workload of developing teaching materials is large, but they can be used multiple times.
Technology integration	Integration-related outcome expectations	In some cases, realia are as convenient and effective as IT tools to facilitate students’ learning.
		I rarely think about how to deeply integrate IT tools with my teaching activities.
	Technology-teaching integration competencies	I can master new technologies, but effectively integrating them into my teaching content is indeed a problem.
		Technical training mainly focuses on theories and skills, with little concern for integration.
		I think I cannot achieve high-quality integration between my content knowledge and IT.

The conceptualization and definition of the technostress creators were developed based on the interview data and existing relevant literature. Specifically, unreliability refers to the degree to which teachers perceive the IT tools used at work as unreliable (Ayyagari et al., 2011). IT may behave inconsistently due to issues such as system failures, slow response, and breakdowns, consequently making teachers feel worried and anxious. Role conflict refers to the degree to which teachers perceive inconsistency of various expectations or conflicts between teachers’ capabilities or beliefs and work requirements due to IT use at work (Guimaraes and Igbaria, 1992; Liu et al., 2019). Although teachers can handle multiple tasks at a time with IT, they may be unsure what to prioritize (e.g., fulfilling ICT demands or focusing on content teaching activities) or lack the capabilities needed to perform the roles. Teaching interruptions refer to the degree to which teachers perceive their teaching activities as being interrupted or distracted by events associated with IT (e.g., incompatibility, unexpected information) (Fletcher et al., 2018; Puranik et al., 2019). As teaching is a flow and interactional activity, unexpected IT events may distract teachers or interrupt teaching, consequently lowering the teaching quality. Information overload refers to the degree to which teachers feel overwhelmed by too much information (Matthes et al., 2019). Generally, teachers have to deal with enormous amounts of information in their daily work. Work overload refers to the degree to which teachers perceive that, due to IT use at work, they have to work more quickly than ever before (Tarafdar et al., 2011). In some situations, IT does not help teachers to work faster or be more productive; conversely, it creates extra work (e.g., learning skills about IT continuously), repetitive work (e.g., working on different applications to fulfil a task), or time-consuming work (e.g., developing digital teaching materials). Technology integration describes the situation where teachers feel that they have insufficient expectancy or competence to integrate IT tools into content knowledge teaching activities (Cheng et al., 2020; Kopcha, 2012). Several teachers reported that becoming familiar with the emerging technology is not difficult, but understanding its usefulness and integrating it with content teaching is a real problem.

## 4 Phase 2

We performed a quantitative study to further understand how technostress creators identified in Phase 1 impact teachers’ IT use behaviors. Guided by the challenge-hindrance framework (Shi et al., 2024), we developed a research model to examine the associations between technostress creators, cognitive appraisal, and IT use behaviors. Empirical data were collected to verify the hypotheses.

### 4.1 Hypotheses development

This study is grounded in the transactional theory of stress (Lazarus and Folkman, 1984). It proposes that stress reactions do not stem directly from objective environmental stimuli. Instead, individuals’ primary cognitive appraisal determines whether a given stress-evoking event is perceived as a challenge or a threat. Recent technostress studies (e.g., Maier et al., 2021) have imported this challenge/threat appraisal (TA) logic from transactional stress theory to interpret the dual nature of technostress creators. We use this appraisal logic to build our hypotheses about how distinct technostress creators trigger challenge or threat appraisals and further shape teachers’ IT-use behaviors.

The transactional theory of stress introduced by Lazarus and Folkman (1984) is a widely adopted theoretical framework to study technostress (Nascimento et al., 2024; Shirish et al., 2021; Tarafdar et al., 2019; Zhao et al., 2020). According to this theory, whether the IT-related environmental conditions will lead to technostress depends closely on individuals’ cognitive appraisal outcomes (i.e., challenge or threat). In particular, challenge appraisal describes individuals viewing technostress creators as beneficial and rewarding, and overcoming them can bring about personal growth, while threat appraisal depicts individuals considering technostress creators as harmful and negative, and working with them will hinder goal achievement (Maier et al., 2021; Shi et al., 2024). Only when individuals evaluate an IT-related event as a threat will they feel strained (Maier et al., 2021).

Several scholars have argued that technostress creators have a dual nature, consisting of both negative and positive sides (Nascimento et al., 2024; Tarafdar et al., 2019). The interview data from Phase 1 also illuminated that some technostress creators are beneficial, providing opportunities for teachers to improve their work performance. Thus, teachers may appraise a specific technostress creator as a threat or challenge, or both. For instance, in terms of information overload, according to cognitive load theory, individuals’ capacities to encode, store, and retrieve information are limited (Lang, 2000; Lang et al., 2013). Thus, being exposed to excessive information may pose a threat to teachers because their limited capacities would be significantly consumed (Shin and Shin, 2016). However, checking the enormous amount of information enables teachers to manage their work dynamics and fulfil work demands promptly. In this situation, information overload is a challenge for teachers. As for role conflict, the inconsistent requirements from varying role expectations may make individuals feel annoyed and exhausted (Liu et al., 2019), threatening their professional growth. However, with IT tools, teachers can handle multiple tasks at the same time; their working efficiency and productivity would be enhanced if resources and energy are allocated properly. In this regard, role conflict seems to be a challenge. Therefore, it is reasonable to assume that teachers may appraise technostress creators as both challenges and threats. Accordingly, we formulated the following hypotheses:

*H1*: Teachers will appraise unreliability (H1a), role conflict (H1b), teaching interruption (H1c), information overload (H1d), work overload (H1e), and technology integration (H1f) as challenges.

*H2*: Teachers will appraise unreliability (H2a), role conflict (H2b), teaching interruption (H2c), information overload (H2d), work overload (H2e), and technology integration (H2f) as threats.

Individuals’ cognitive appraisal of environmental conditions would determine the damaging or beneficial outcomes (Lazarus, 1966). Previous studies on technostress also proposed and confirmed that challenge appraisal always leads to positive outcomes, whereas threat appraisal is likely to result in negative outcomes (e.g., Zhao et al., 2020). For teachers, when they evaluate a technostress creator as a threat that leads to personal loss, constraint, or harm, they presumably respond to it with psychological strain, for example, reduced satisfaction or a high intention to reject IT. Conversely, if teachers evaluate a technostress creator as a challenge that leads to personal growth, freedom, or benefit, they may respond to it with enthusiasm, for example, increased satisfaction or innovative use. To understand the impact of technostress creators on teachers, this study focuses on two typically identified IT use behaviors—innovative use and avoidant use—which were considered important for individuals’ and organizations’ productivity (Bala and Venkatesh, 2016; Roberts et al., 2016). In this study, innovative use describes the situation in which teachers used IT tools in a novel way to facilitate teaching and learning, which may be triggered by challenge appraisal (Maier et al., 2021). Avoidant use describes the situation in which teachers tend to complete work tasks without IT or avoid using IT as much as possible, which may be linked to threat appraisal (Maier et al., 2021; Yu et al., 2023). Accordingly, we formulated the following hypotheses:

*H3*: Challenge appraisal outcomes will positively predict teachers’ innovative use of IT (H3a), but negatively predict avoidant use of IT (H3b).

*H4*: Threat appraisal outcomes will negatively predict teachers’ innovative use of IT (H4a), but positively predict avoidant use of IT (H4b).

### 4.2 Measurement/instruments

A questionnaire consisting of two parts was developed for data collection. The first part was designed to collect demographic characteristics of teachers, including four single-choice questions on gender, age, teaching stage, and teaching subject, and one multiple-choice question about the IT tools commonly used at work. The second part consisted of 27 items measuring the constructs in the research model, which were revised from existing studies and presented on a 5-point Likert scale. A pilot test was performed with 10 teachers, and slight wording revisions were made based on their feedback. Details of the instruments were provided in Appendix D.

### 4.3 Data collection

A total of 309 teachers completed the online survey developed on Wenjuanxing^1^, the most popular online survey website in China. A statement about the purpose of the study and data use was sent with the questionnaire link, guaranteeing voluntary and anonymous participation. Among these responses, 44 did not pass the screening criterion “If you are answering the question seriously, please select ‘strongly agree’ for this question”. After removing these responses, we conducted a thorough screening of the remaining responses and found no outliers, resulting in a valid sample size of 265. As for the sample size for structural equation modeling, scholars suggested that the ratio of sample size to the number of items measured should be greater than 5:1 (Hair et al., 2006); a sample size larger than 200 is appropriate (Kline, 2011). The sample size in this study satisfied both thresholds. Details of the sample are presented in Table 3.

**Table 3:** Demographic information of respondents.

Characteristic	Category	Number	Percentage
Gender	Female	164	61.89
	Male	101	38.11
Age	Below 30	70	26.42
	31–40	96	36.23
	41–50	74	27.92
	Above 50	25	9.43
Teaching stage	Kindergarten	20	7.55
	Primary school	87	32.83
	Middle school	84	31.70
	University	74	27.92
Teaching subject	Natural sciences	50	18.87
	Social sciences	82	30.94
	Languages	77	29.06
	Engineering and technology	56	21.13
Commonly used IT tools at work (multiple-choice)	Multimedia teaching equipment	264	99.62
	Document management tools	240	90.57
	Social media apps	231	87.17
	Online teaching system	172	64.91
	Other subject-related tools	71	26.79

### 4.4 Data analysis and results

#### 4.4.1 Common method bias testing

Common method bias testing was performed using Harman’s single-factor test. The results showed that the variance explained by the first factor with an eigenvalue greater than 1 was 26.92%, less than the 40% criterion (Podsakoff et al., 2003), indicating that there was no significant common method bias in the data.

#### 4.4.2 The measurement model

According to Schumacker and Lomax (2004), standardized factor loadings of the items should not be below 0.50. The item WO1 was removed because of its low factor loading. The factor loadings of the remaining items were all above 0.50 (Schumacker and Lomax, 2004); composite reliability (CR) and average variance extracted (AVE) coefficients are greater than 0.70 and 0.50 (Table 4), respectively, above the thresholds proposed by Fornell and Larcker (1981). Thus, convergent validity is acceptable. In terms of discriminant validity, the results indicated that, for each variable, the square root of AVE is greater than the correlation coefficients (Table 5), suggesting sufficient discriminant validity (Fornell and Larcker, 1981). Moreover, the fit of the measurement model is also good (Hu and Bentler, 1999), with chi-square/degrees of freedom (CMIN/DF) = 1.449, root mean square residual (RMR) = 0.034, goodness-of-fit index (GFI) = 0.907, Tucker–Lewis index (TLI) = 0.973, comparative fit index (CFI) = 0.978, and root mean square error of approximation (RMSEA) = 0.041.

**Table 4:** Results of the reliability and validity analysis.

Variables	Items	Loadings	CR	AVE	*α*	Mean	SD
Unreliability	UNR1	0.875	0.924	0.803	0.923	2.644	0.874
	UNR2	0.924					
	UNR3	0.888					
Role conflict	RC1	0.599	0.827	0.620	0.812	2.613	0.770
	RC2	0.841					
	RC3	0.891					
Teaching interruption	TI1	0.781	0.877	0.704	0.872	2.745	0.952
	TI2	0.883					
	TI3	0.849					
Information overload	IO1	0.870	0.908	0.766	0.907	3.821	0.733
	IO2	0.901					
	IO3	0.854					
Work overload	WO2	0.931	0.870	0.771	0.866	3.557	0.861
	WO3	0.822					
Technology integration	TIG1	0.904	0.941	0.890	0.768	2.817	0.773
	TIG2	0.981					
Challenge appraisal	CA1	0.899	0.962	0.893	0.961	3.860	0.787
	CA2	0.980					
	CA3	0.955					
Threat appraisal	TA1	0.904	0.970	0.914	0.969	2.091	0.929
	TA2	0.981					
	TA3	0.982					
Innovative use	INU1	0.723	0.856	0.753	0.836	3.298	0.804
	INU2	0.992					
Avoidant use	AU1	0.887	0.836	0.718	0.832	2.111	0.782
	AU2	0.806					

**Table 5:** Results of the discriminant validity analysis.

Variables	1	2	3	4	5	6	7	8	9	10
1 Unreliability	**0.896**									
2 Role conflict	0.196	**0.787**								
3 Teaching interruption	0.204	0.276	**0.839**							
4 Information overload	0.224	0.176	0.191	**0.875**						
5 Work overload	0.025	−0.259	−0.164	0.161	**0.878**					
6 Technology integration	0.003	−0.221	0.109	0.034	0.340	**0.943**				
7 Challenge appraisal	0.087	−0.305	−0.240	0.033	0.512	0.603	**0.945**			
8 Threat appraisal	0.252	0.307	0.477	0.059	−0.382	0.080	−0.380	**0.956**		
9 Innovative use	0.025	0.109	0.078	−0.123	0.400	0.239	0.384	0.105	**0.868**	
10 Avoidant use	0.206	0.366	0.196	0.041	−0.386	−0.259	−0.497	0.447	−0.232	**0.847**

The bold diagonal values are the square root of AVE.

#### 4.4.3 The structural model

The results of structural equation modeling are presented in Table 6. It was found that work overload (*β* = 0.391, *p* < 0.0001) and technology integration (*β* = 0.592, *p* < 0.0001) are positively associated with challenge appraisal, while unreliability (*β* = −0.027, *p* > 0.05), role conflict (β = −0.062, *p* > 0.05), and information overload (*β* = −0.084, *p* > 0.05) have no significant association with challenge appraisal, supporting H1e and H1f and rejecting H1a, H1b, and H1d. However, teaching interruption (−0.126, *p* < 0.05) is negatively associated with challenge appraisal, which is contrary to H1c. Furthermore, unreliability (*β* = 0.172, *p* < 0.0001), role conflict (*β* = 0.132, *p* < 0.05), and teaching interruption (*β* = 0.422, *p* < 0.0001) positively predict threat appraisal, supporting H2a, H2b, and H2c. Information overload (*β* = −0.007, *p* > 0.05) and technology integration (*β* = 0.070, *p* > 0.05) have no significant effect on threat appraisal, rejecting H2d and H2f. Work overload (*β* = −0.370, *p* < 0.0001) negatively predicts threat appraisal, contrary to H2e. The model accounted for 53.1% of variance in challenge appraisal and 36.7% of the variance in threat appraisal. The model explained 33.4% of the variance in avoidant use through challenge appraisal (*β* = −0.390, *p* < 0.001) and hindrance appraisal (*β* = 0.365, *p* < 0.001), which supported H3b and H4b. Additionally, challenge appraisal (*β* = 0.391, *p* < 0.001) positively predicts innovative use, while threat appraisal (*β* = −0.009, *p* > 0.05) had no significant effect on innovative use, supporting H3a and rejecting H4a. Furthermore, the *R*^2^ value of innovative use is 15.4%. The study model with its standardized coefficients is shown in Figure 2.

**Table 6:** The results of the hypothesis testing.

Hypotheses	Paths	*β*	*B*	S. E.	*T*-value	Supported?
H1a	Unreliability → Challenge appraisal	−0.027	−0.021	0.039	−0.535	No
H1b	Role conflict → Challenge appraisal	−0.062	−0.080	0.066	−1.210	No
H1c	Teaching interruption → Challenge appraisal	−0.126	−0.095	0.039	−2.460*	Inversely
H1d	Information overload → Challenge appraisal	−0.084	−0.069	0.041	−1.661	No
H1e	Work overload → Challenge appraisal	0.391	0.394	0.056	6.978***	Yes
H1f	Technology integration → Challenge	0.592	0.592	0.069	8.569***	Yes
H2a	Unreliability → Threat appraisal	0.172	0.166	0.052	3.189**	Yes
H2b	Role conflict → Threat appraisal	0.132	0.208	0.089	2.35*	Yes
H2c	Teaching interruption → Threat appraisal	0.422	0.391	0.056	7.036***	Yes
H2d	Information overload → Threat appraisal	−0.007	−0.007	0.054	−0.122	No
H2e	Work overload → Threat appraisal	−0.370	−0.457	0.073	−6.274***	Inversely
H2f	Technology integration → Threat appraisal	0.070	0.086	0.071	1.217	No
H3a	Challenge appraisal → Innovative use	0.391	0.353	0.079	4.472***	Yes
H3b	Challenge appraisal → Avoidant use	−0.390	−0.393	0.063	−6.271***	Yes
H4a	Threat appraisal → Avoidant use	0.365	0.299	0.050	5.936***	Yes
H4b	Threat appraisal → Innovative use	−0.009	−0.007	0.042	−0.156	No

***p < 0.001, **p < 0.01, *p < 0.05.

**Figure 2:**
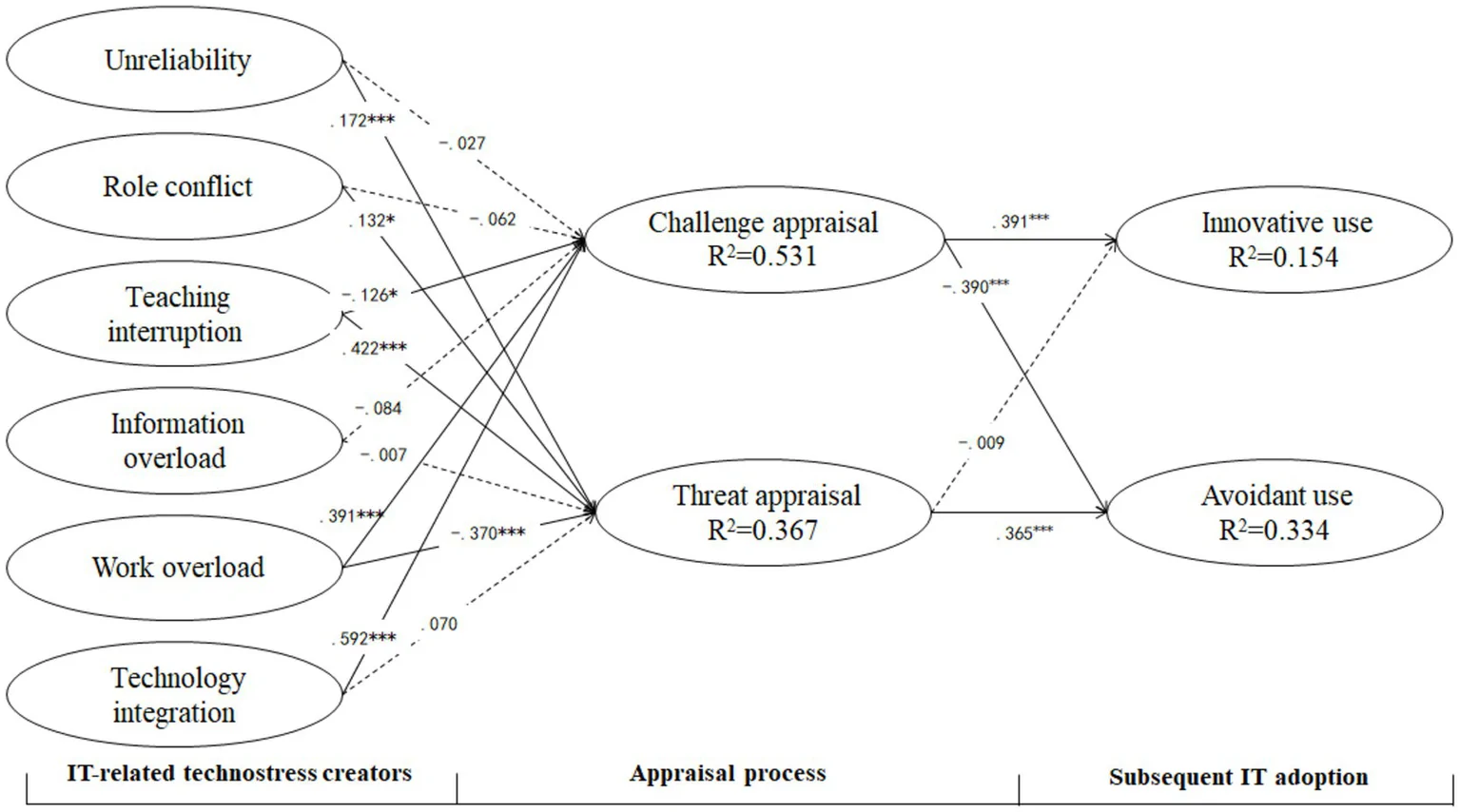
The research model with its standardized coefficients. *p < 0.05; ***p < 0.001.

#### 4.4.4 Indirect effects of technostress creators on IT-use behaviors

To examine the mediating effects of cognitive appraisal between technostress creators and teachers’ IT-use behaviors, we conducted bias-corrected bootstrap mediation analysis with 5,000 resamples at the 95% confidence level. Table 7 presents the standardized total indirect effects, together with 95% bias-corrected bootstrap lower and upper confidence intervals (LLCI, ULCI). Mediation is statistically significant when the 95% CI excludes zero. The reported total indirect effects combine the indirect influences transmitted via both challenge appraisal and threat appraisal. The results revealed that indirect effects are significant for five pathways. Specifically, teaching interruption exerts a significant positive indirect effect on avoidant use (*β* = 0.154). Work overload showed a significant positive indirect effect on innovative use (*β* = 0.142) and a significant negative indirect effect on avoidant use (*β* = −0.291). Similarly, technology integration yielded a significant positive indirect effect on innovative use (*β* = 0.209) and a significant negative indirect effect on avoidant use (*β* = −0.207). The remaining seven indirect pathways were non-significant because their 95% CI contained zero. These results indicate that only certain technostress creators can shape teachers’ innovative and avoidant IT-use behaviors through their cognitive appraisal.

**Table 7:** The indirect effects of technostress creators on IT-use behaviors.

Indirect paths	*β*	LLCI	ULCI	*P*	Significant?
Unreliability → appraisal → Innovative use	−0.008	−0.045	0.039	0.705	No
Unreliability → appraisal → Avoidant use	0.058	−0.010	0.122	0.098	No
Role conflict → appraisal → Innovative use	−0.030	−0.093	0.035	0.306	No
Role conflict → appraisal → Avoidant use	0.094	−0.010	0.215	0.076	No
Teaching interruption → appraisal → Innovative use	−0.036	−0.082	0.010	0.099	No
Teaching interruption → appraisal → Avoidant use	0.154	0.085	0.229	0.000	Yes
Information overload → appraisal → Innovative use	−0.024	−0.090	0.011	0.185	No
Information overload → appraisal → Avoidant use	0.025	−0.038	0.095	0.386	No
Work overload → appraisal → Innovative use	0.142	0.059	0.283	0.000	Yes
Work overload → appraisal → Avoidant use	−0.291	−0.502	−0.159	0.000	Yes
Technology integration → appraisal → Innovative use	0.209	0.095	0.363	0.000	Yes
Technology integration → appraisal → Avoidant use	−0.207	−0.373	−0.078	0.002	Yes

## 5 Discussion

This study strives to obtain a systematic understanding of teachers’ technostress associated with IT use at work through a mixed-methods design. The main findings are as follows:

The qualitative findings established six technostress creators: unreliability, role conflict, teaching interruption, work overload, information overload, and technology integration. The results confirmed that teachers experience unique technological conditions that create strain. First, teachers’ IT use creates interruption pressure because teaching requires highly focused attention and emotion, whereas the characteristics of technology (e.g., continuous connectivity, incompatibility between devices) enable teaching interruption at any time. Second, teachers’ IT use creates technology integration pressure. Technology integration is innovative and situational; teachers cannot directly copy the model of others but must explore an effective way according to the specific teaching situation. Furthermore, the interview data also illustrated that not all technostress creators are harmful; some of them are “good” or “beneficial,” guiding the quantitative study.

The quantitative findings suggested that unreliability, role conflict, and teaching interruption are harmful because teachers appraise them as threatening. These findings indicated that for teachers, unreliability, role conflict, and teaching interruption are the negative aspects of IT. Working with unreliable technologies puts individuals at high risk of experiencing strain and awkwardness (Califf et al., 2020), especially for teachers who have to work in front of a large group of students. As for role conflict, previous studies (e.g., Chou and Chou, 2021) and our interview data revealed that the use of IT makes the teachers’ roles more diverse, exacerbating role conflicts. Teachers have to deal with multiple tasks simultaneously due to the various role expectations, some of which are even beyond their expertise and capabilities, which probably results in burnout and exhaustion. Interruptions to work were confirmed to be negative for individuals’ work performance and emotions (Baethge and Rigotti, 2013; Jett and George, 2003). In the teaching context, the unexpected disturbances associated with IT would easily divert the attentional focus of both teachers and students and even lead to the suspension of ongoing teaching activities; consequently, teachers may fail to perform as planned.

However, different from the three above threat-related technostress creators, work overload plays a dual role in teachers’ cognitive appraisal process. On the other hand, work overload positively predicts challenge appraisal. Although IT brings extra workload, teachers interpret increased workload as rewarding opportunities for professional growth; overcoming these additional tasks can help them improve digital teaching competence, which aligns with prior teacher technostress studies (Li and Wang, 2021; Wang and Zhao, 2023). On the other hand, work overload shows a significant negative effect on threat appraisal, meaning higher work overload does not trigger teachers’ hindrance perception. Rather than viewing the heavier workload brought by IT as a harmful threat, teachers in our sample tend to frame such extra demands as achievable professional challenges. We also found that technology integration is another challenge for teachers. The gap in today’s classroom is not technology availability or operation, but appropriate technology integration for instructional purposes (Kopcha, 2012). Although teachers have to eliminate barriers to effectively incorporate IT into instruction, they view it as rewarding because effective technology integration has become a crucial requirement for a quality teacher (Çebi et al., 2022).

An unexpected finding is that information overload seems neither a challenge nor a threat to teachers. That is, information overload has no significant effect on teachers’ technostress appraisal. This is contrary to previous studies, which reported that information overload positively predicts users’ exhaustion and stress (Pang and Ruan, 2023; Shin and Shin, 2016). We provide some possible explanations for this result. First, digital information has become a routine part of teachers’ work, enabling smooth and effective communication among relevant parties (e.g., parents, students, teachers, and administrators). Second, teachers are a well-educated group who may have sufficient capabilities to deal with the massive amounts of information through effective methods (e.g., quick browsing and timed checking), balancing information management with other work. Third, from a measurement perspective, information overload obtained the highest mean score (*M* = 3.82) among the six technostress creators, which raises the possibility of ceiling effects. High average scores may restrict variance and attenuate its statistical relationships with appraisal variables. In addition, social desirability bias might be present; teachers may be reluctant to report being overwhelmed by work-related digital information, leading to response compression.

It was found that teachers’ challenge appraisal positively predicts innovative use but negatively predicts avoidant use, whereas threat appraisal positively predicts avoidant use. These findings were partly consistent with Yu et al. (2023), who also reported positive associations between challenge appraisal and innovative use and hindrance appraisal and avoidant use; the study included 439 employees from various industries. Our study further confirms that these relationships also exist in the context of teachers’ IT use. When teachers interpret IT-specific events or properties as opportunities to learn and grow, they are likely to overcome difficulties to use technologies innovatively to facilitate teaching and learning. Conversely, if teachers consider IT use to disrupt their work habits and hinder their personal achievements, they may try to avoid using IT as much as possible.

## 6 Contributions, implications, and limitations

### 6.1 Contributions

This study has several theoretical contributions. First, contrary to existing studies that solely employed quantitative methods to test the five typical technostress creators among teachers, our study identified and conceptualized context-specific technostress creators associated with IT use among teachers through in-depth interviews. The findings suggested that teachers experience unique technological conditions that create strain, offering a theoretical basis for further work related to teachers’ technostress. Furthermore, the findings reminded researchers to consider the domain-specific typologies of technostress creators. Thus, there is a necessity to understand the precise technostress creators within a given IT use context because the events triggering technostress may vary across disciplines. Finally, our study illustrated how technostress creators influence teachers’ IT use by evaluating the association between technostress creators, technostress appraisal, and IT use intentions, expanding the literature on teachers’ IT use.

### 6.2 Implications

The findings provided valuable practical insights for school administrators and teachers. For school administrators, special attention should be paid to unreliability, role conflict, and teaching interruption because they pose threats to teachers. First, enhancing the stability and reliability of IT can reduce teachers’ anxiety and uncertainty (Nascimento et al., 2024). For common technical problems, solutions can be presented in a prominent position through a combination of graphics and text. Second, to mitigate teachers’ perception of role conflict, effective administrative support, such as reducing unnecessary work-related complications, simplifying workflow, and removing conflicting work requirements, is favorable and enables teachers to focus on more core tasks. Third, in the digital age, working without interruption has become a luxury (Tams et al., 2020). To limit teachers’ exposure to interruptions, school administrators should provide teachers with greater work control (e.g., method control and criteria control) to accomplish their work. Finally, technology integration was considered a challenging opportunity, facilitating the teaching and learning process. Several interviewees in our study reported that the successful integration experience of subject teachers is very inspiring for their own use of IT. Thus, in addition to providing technical training, a similar workshop aiming to share successful experiences in applying the newly introduced technology to specific teaching content will enhance teachers’ technology integration.

For teachers, with the ongoing digitalization of education, it is inevitable that teachers will encounter technostress. Our findings suggested that teachers’ challenge and threat appraisal outcomes influence subsequent IT use in different ways, highlighting the importance of the cognitive appraisal process. Hence, teachers need to adjust their behaviors to changing teaching environments and view the requirements associated with IT as challenges that contribute to their personal growth and achievements. They can fulfil the IT tasks by taking effective measures, such as familiarizing themselves with the technologies introduced and seeking technical help or social support proactively. Furthermore, since psychological capital can help individuals cope with stress (Zhang et al., 2019), teachers can manage technostress creators better by cultivating positive psychological qualities such as self-efficacy, hope, and optimism.

### 6.3 Limitations

First, the sample size of the empirical sample is relatively small, which may limit the generalizability of our findings. Subsequent studies can verify the relationships with a larger sample. Second, our data were collected from Chinese teachers, without considering teachers from other countries or regions. Since the espoused cultural values also account for the variation in technostress phenomena (Krishnan, 2017), future work can replicate this study with teachers from different cultures to obtain a more comprehensive understanding of technostress creators in the education field. Third, this study did not examine the effects of technostress inhibitors (e.g., technical support provision and technology involvement facilitation), which were considered closely related to individuals’ technostress appraisal and subsequent outcomes (Shi et al., 2024; Tarafdar et al., 2011). Further studies can explore the boundary effects of technostress inhibitors on the relationships in this study. Furthermore, several latent constructs rely on two-item measurements, constraining the content validity. Future research should develop and validate expanded multi-item scales for these constructs to achieve more comprehensive content coverage. In the qualitative analysis, member-checking with the original interview respondents could not be implemented in this study. Subsequent studies in this domain can seek to incorporate respondent validation to further strengthen interpretive trustworthiness.

## 7 Conclusion

This study contributes to existing literature by paying distinctive attention to context-specific technostress creators among teachers as well as by evaluating their impact on IT use behaviors. The findings provide evidence for school administrators and teachers to manage technostress effectively and purposefully. Future studies involving teachers from other countries and analyzing the effects of technostress inhibitors appear to be promising.

## Data Availability

The raw data supporting the conclusions of this article will be made available by the authors, without undue reservation.

## Footnotes

1
https://www.wjx.cn/
