# Supplementary material for: Understanding teachers’ technostress creators due to IT use at work: a mixed-methods study
Source: Front Psychol. 2026 Oct 2;17:1887403. doi: 10.3389/fpsyg.2026.1887403 (PMC13672583; doi:10.3389/fpsyg.2026.1887403)
Supplement: Supplementary file 1 [file Table_1.DOCX]

Appendix A. Comparison of existing typical technostress creators research

| **Researchers** | **Research context** | **Technostress creators** | **Limitations for education context** |
| --- | --- | --- | --- |
| Tarafdar et al. (2007) | IT industry employees | techno‑overload, techno‑complexity, techno‑invasion, techno‑insecurity, techno‑uncertainty | Developed for IT professionals; does not consider teaching‑specific events such as classroom interruptions, curriculum‑technology integration demands |
| Shi et al. (2024) | Hospitality employees | persistence,  role conflict,  work connectivity,  visibility,  emotional interruption | Focused on hospitality service work; lacks considerations for instructional and curriculum‑related requirements |
| Maier et al. (2022) | Users under trial‑period IT implementation | perceived difficulty of use,  unreliability,  monitoring,  incompatibility,  privacy concerns,  transition costs,  personalization complexity,  habit disruption | Focuses on short‑term new‑system adoption, doesn’t reflect teachers’ long‑term daily IT‑enabled teaching practice |

Appendix B. Details of the interviewees

| **Number** | **Gender** | **Age** | **Years of teaching** | **Teaching subject** | **Teaching stage** |
| --- | --- | --- | --- | --- | --- |
| 1 | Male | 30 | 6 | Social sciences | Middle school |
| 2 | Male | 25 | 2 | STEM | Primary school |
| 3 | Female | 39 | 11 | Music | Kindergarten |
| 4 | Female | 31 | 6 | STEM | Primary school |
| 5 | Female | 28 | 4 | Social sciences | Primary school |
| 6 | Female | 33 | 8 | Social sciences | Middle school |
| 7 | Male | 35 | 12 | Languages | Middle school |
| 8 | Female | 26 | 2 | Social sciences | University |
| 9 | Female | 51 | 30 | Social sciences | University |
| 10 | Female | 24 | 2 | Languages | Middle school |
| 11 | Male | 43 | 15 | Social sciences | Middle school |
| 12 | Male | 27 | 4 | Natural sciences | University |
| 13 | Male | 27 | 3 | Natural sciences | University |
| 14 | Male | 32 | 9 | Languages | University |
| 15 | Female | 29 | 5 | Languages | University |
| 16 | Female | 42 | 18 | Engineering technology | University |
| 17 | Female | 34 | 12 | Engineering technology | University |
| 18 | Female | 26 | 3 | Social sciences | Primary school |
| 19 | Female | 44 | 15 | STEM | Primary school |
| 20 | Female | 54 | 33 | STEM | Middle school |

Appendix C. Outline of semi-structured interviews

**Basic information**

Gender: □ Female □ Male

Educational level: □ Bechelor or below □ Postgraduate or above

Age:_______

Years of teaching:_______

Teaching subject:_______

Teaching stage: □ Kindergarten □ Primary school □ Middle school □ University

**Instructions**

Our study strives to understand teachers’ IT use at work. The following questions have no correct or incorrect answers. Please answer them based on your experience. We promise that your interview data will be strictly confidential and used only for research purposes.

**Open-ended questions**

- Please briefly describe your work responsibility.
- What are the commonly used IT tools in work? How often do you use them?
- Do you feel any stress when using IT at work? If yes, please describe your feelings under such stress.
- What characteristics of IT or events associated with IT do you think contribute to such stress?
- Do you think the stress initiated by IT use has any negative impacts on your subsequent IT use? Why or why not?
- Do you think the stress initiated by IT use has any positive impacts on your subsequent IT use? Why or why not?
- Do you have any other issues/feelings that you want to share about your use of IT at work?

Appendix D. Survey items

| **Item** |  | **Reference** |
| --- | --- | --- |
| **Unreliability** | | Ayyagary et al. (2011) |
| At work, I sometimes felt like | |  |
| UNR1. the features provided by IT are undependable. | |  |
| UNR2. the capabilities provided by IT are unreliable. | |  |
| UNR3. IT doesn’t behave in a highly consistent way. | |  |
| **Role conflict** | | Shi et al. (2024) and Liu et al., (2019) |
| RC1.I get tasks related to IT that I do not have adequate resources to complete. | | |
| RC2.People in our school have incompatible expectations of my IT use behaviours. | | |
| RC3.I spend time on IT working on pointless things. | | |
| **Teaching interruption** Shi et al. (2024) | | |
| TI1.Sometimes my teaching activities were interrupted by IT. | | |
| TI2.During classroom teaching, dealing with teaching interruptions initiated by IT is time-consuming. | | |
| TI3.Sometimes I have to stop the ongoing teaching activities to respond to the interruptions initiated by IT. | | |
| **Information overload** | | Müller et al. (2023) |
| Due to my work, I sometimes felt like | | |
| IO1.not being able to effectively process all the information I receive. | | |
| IO2.being overwhelmed by the information I receive. | | |
| IO3.being afraid of having missed/forgotten a vital information I received. | | |
| **Work overload** | | Tarafdar et al. (2011) |
| WO1.I was forced by IT to do more work than I can handle. | | |
| WO2.I was forced by IT to work with very tight time schedules. | | |
| WO3.I have a higher workload because of the use of IT. | | |
| **Technology integration** | | Cheng et al., 2020 |
| TIG1.It is not easy to achieve high-quality integration between my knowledge content and IT. | | |
| TIG2.It is not easy to use IT to involve learners in self-assessment and goal setting. | | |
| **Challenge appraisal** | | LePine et al. (2016) |
| CA1.Working to fulfill the IT demands of work improves my personal growth. | | |
| CA2.The IT demands of work promote my personal accomplishment. | | |
| CA3. Working to fulfill the IT demands of work helps to improve my well-being | | |
| **Threat appraisal** | | LePine et al. (2016) |
| TA1.Working to fulfill the IT demands of work constrains my personal growth. | | |
| TA2.The IT demands of work hinders my personal accomplishment. | | |
| TA3. The IT demands of work threats my well-being | | |
| **Innovative use** | | Yu et al., (2023) |
| INU1.I have discovered new uses of IT to enhance my work performance. | | |
| INU2.I have used IT in novel ways to support my work. | |  |
| **Avoidant use** | | Yu et al., (2023) |
| AU1.I try to avoid IT as much as I can. | |  |
| AU2.I find ways to complete most of my daily work activities without using IT. | | |
